# Novel Nucleotide and Amino Acid Covariation between the 5′UTR and the NS2/NS3 Proteins of Hepatitis C Virus: Bioinformatic and Functional Analyses

**DOI:** 10.1371/journal.pone.0025530

**Published:** 2011-09-28

**Authors:** Hung-Yu Sun, Nai-Ying Ou, Shainn-Wei Wang, Wen-Chun Liu, Ting-Fang Cheng, Shiou-Jiuan Shr, Koun-Tem Sun, Ting-Tsung Chang, Kung-Chia Young

**Affiliations:** 1 Institute of Basic Medical Sciences, College of Medicine, National Cheng Kung University, Tainan, Taiwan; 2 Department of Medical Laboratory Science and Biotechnology, College of Medicine, National Cheng Kung University, Tainan, Taiwan; 3 Institute of Molecular Medicine, College of Medicine, National Cheng Kung University, Tainan, Taiwan; 4 Department of Biotechnology, College of Applied Science, MingDao University, ChangHua, Taiwan; 5 Department of Information and Learning Technology, Science and Engineering College, National University of Tainan, Tainan, Taiwan; 6 Department of Internal Medicine, College of Medicine, National Cheng Kung University, Tainan, Taiwan; 7 Center of Infectious Disease and Signaling Research, National Cheng Kung University, Tainan, Taiwan; University of Vermont, United States of America

## Abstract

Molecular covariation of highly polymorphic viruses is thought to have crucial effects on viral replication and fitness. This study employs association rule data mining of hepatitis C virus (HCV) sequences to search for specific evolutionary covariation and then tests functional relevance on HCV replication. Data mining is performed between nucleotides in the untranslated regions 5′ and 3′UTR, and the amino acid residues in the non-structural proteins NS2, NS3 and NS5B. Results indicate covariance of the 243^rd^ nucleotide of the 5′UTR with the 14^th^, 41^st^, 76^th^, 110^th^, 211^th^ and 212^th^ residues of NS2 and with the 71^st^, 175^th^ and 621^st^ residues of NS3. Real-time experiments using an HCV subgenomic system to quantify viral replication confirm replication regulation for each covariant pair between 5′UTR_243_ and NS2-41, -76, -110, -211, and NS3-71, -175. The HCV subgenomic system with/without the NS2 region shows that regulatory effects vanish without NS2, so replicative modulation mediated by HCV 5′UTR_243_ depends on NS2. Strong binding of the NS2 variants to HCV RNA correlates with reduced HCV replication whereas weak binding correlates with restoration of HCV replication efficiency, as determined by RNA-protein immunoprecipitation assay band intensity. The dominant haplotype 5′UTR_243_-NS2-41-76-110-211-NS3-71-175 differs according to the HCV genotype: G-Ile-Ile-Ile-Gly-Ile-Met for genotype 1b and A-Leu-Val-Leu-Ser-Val-Leu for genotypes 1a, 2a and 2b. In conclusion, 5′UTR_243_ co-varies with specific NS2/3 protein amino acid residues, which may have significant structural and functional consequences for HCV replication. This unreported mechanism involving HCV replication possibly can be exploited in the development of advanced anti-HCV medication.

## Introduction

Co-evolution was initially defined as covarying genetic adaptation between species in an environment. More recently, the concept of covariation has been extended to covarying amino acids at the molecular level of proteins, mostly involving the coordinated change of certain amino acid residues in response to the change of other amino acid residues to maintain biologically relevant structures and functions [1]. Amino acid covariation is commonly observed in polymorphic viruses. Such behavior may result in compensatory mutations by which an evolving mutation with reduced fitness can be rescued. It is well known that triple mutations of Ile63Met, Val189Ile and Glu396Gly partially restore the enzymatic activity of a Trp229Tyr reverse transcriptase mutant of the human immunodeficiency virus type 1 [2]. As an alternative example, Leu180Met and Val173Leu mutations may enhance the replicative efficiency of a reverse transcriptase Tyr-Met-Asp-Asp motif mutant of the hepatitis B virus [3].

Chronic hepatitis C virus (HCV) infection is a primary factor leading to liver cirrhosis and hepatocellular carcinoma worldwide [4]. Genomic HCV RNA consists of an open reading frame encoding a polypeptide precursor of the sequence NH_2_-core-envelope 1-envelope 2-p7- non-structural (NS) 2-NS3-NS4A-NS4B-NS5A-NS5B-COOH, flanked by the 5′ and 3′ untranslated regions (UTR). An internal ribosome entry site (IRES) within the 5′UTR is essential for translational initiation of the viral RNA [5]. The *cis*-acting elements in the 3′UTR and IRES(-) are indispensable for the RNA replication process [6]–[9]. Among the NS proteins, NS2, NS3 and NS5B perform enzymatic activities that are necessary for the HCV life cycle [10]: (i) NS2 is a cysteine protease responsible for autoprocessing at the NS2-NS3 junction [11], [12]; (ii) NS3 performs dual enzymatic functions as a serine protease for the cleavage of the junctions of NS3/4A, NS4A/4B, NS4B/5A and NS5A/5B and as a RNA helicase/NTPase for unwinding HCV RNA [13], [14]; (iii) NS5B is a RNA-dependent RNA polymerase responsible for replication of the HCV RNA genome [15].

The highly variable HCV genome has been classified into 6 genotypes [16] which affect pathogenesis and therapeutic outcome [17]. Covarying amino acid residues are believed to evolve for persistent viral replication or egression, to be functionally conserved and constrained within certain viral components. Certain compensatory mutations at the protein level have been reported recently [18]-[22]. Coordinated substitutions in NS3 and NS4A affect HCV replication *via* modulation of NS5A phosphorylation [19]. Compensatory mutations in p7 and NS2 restore assembly-defective core protein mutants, whereas chimeric HCV with coordinated mutations in envelope 1, p7, NS2, and NS3 increase the intergenotypic compatibilities for virus assembly and release [20], [22]. More importantly, amino acid covariance networks have been identified to predict the response in HCV patients receiving anti-viral therapy [18], [21]. Such studies underscore the significance of the functional linkage of certain proteins and their covariant amino acid residues for HCV persistency, raising the possibility that molecular covariation can be computationally predicted during persistent infection for diagnosis, prognosis and optimal drug selection.

It is suspected that covariation might involve motifs in the UTRs which regulate HCV genome replication at transcriptional or translational levels and may be essential for persistent HCV. However, no studies have yet addressed covariation between the HCV UTRs and the NS proteins. In the present study, the authors explore the possibility that conserved covariation spots exist between functionally essential nucleotides in the UTRs and the amino acid residues in the 3 enzymatic NS proteins. The association data mining algorithm in the Weka software [23] was used to extract previously unknown and potentially meaningful covariation within the HCV sequences retrieved from the Los Alamos HCV database at the full-length genome level [24]. The functional relevance of the observed covariation sites was then tested in a cell-based HCV replicon system [25], analyzing the effects of either the individual or simultaneous substitutions of those sites with regard to replication efficiency and RNA-protein interactive ability.

## Results

### Bioinformatic analysis - Preparation of 217 full-length HCV genome sequences for association rule data mining

**Figure 1:**
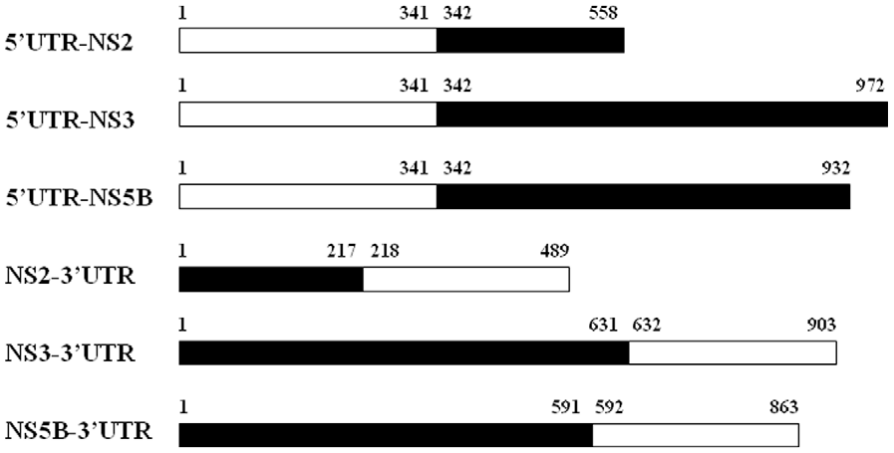
Binary HCV sequence components used for prediction of site-specific covariation between UTRs and NS proteins. Six sequence components for data mining were created by connecting UTR nucleotide segments and amino acid segments of the enzymatic NS proteins, including 5′UTR-NS2, 5′UTR-NS3, 5′UTR-NS5B, NS2-3′UTR, NS3-3′UTR and NS5B-3′UTR. The 5′UTR and 3′UTR contained 341 and 272 nucleotides, respectively; the NS2, NS3 and NS5B contained 217, 631 and 591 amino acid residues, respectively. White bars indicate nucleic acid segments; black bars indicate amino acid segments.

One of the most common applications of association rule mining is ‘market basket’ analysis, i.e. a search is performed from supermarket checkout data for groups of items that occur together in transactions. A similar technique is used in this study, whereby the nucleotides and amino acid positions are considered as attributes in an individual instance. Association rule mining searches for covariation rules between single nucleotides of the UTRs and the amino acid residues of the NS proteins. To this end, 217 full-length HCV genome sequences were downloaded from the Los Alamos HCV sequence database on Nov. 30, 2006. Analysis of the phylogenetic relationships of the HCV sequences indicated that most were clustered into 4 major genotypes, 1a, 1b, 2a and 2b, while the others sporadically presented as 14 minor genotypes (Table S1 and Fig. S1). The individual UTR RNA segments (5′UTR and 3′UTR) and the NS protein segments (NS2, NS3, NS5B) from each full-length genome sequence were retrieved and then connected to create new sequence components (Fig. 1) for covariation analysis. These 6 binary sequence components were input to the Weka software to determine the covariation association between each of the nucleotide sites and the amino acid residues. The unique association rules of these binary sequence datasets are summarized in Table S2. Thirty-nine unique association rules (12 for all genotypes, 11, 2, 8 and 6 for genotypes 1a, 1b, 2a and 2b, respectively) were identified. Results in the set for all genotypes indicate covariance of the 204^th^ nucleotide of the 5′UTR with 3 amino acid residues of the NS3 protein (71, 175 and 621) and the 243^rd^ nucleotide of the 5′UTR with 6 amino acid residues of the NS2 protein (14, 41, 76, 110, 211 and 212) and 3 amino acid residues of the NS3 protein (71, 175 and 621). Since the covariance between 5′UTR_243_ and NS2-14, -41, -76, -110, -211, -212 and NS3-71, -175 and -621 consists of associations involving the largest number of multiple sites, the functional relevance of 5′UTR_243_ in co-variation with the residues in the NS2 and NS3 proteins but not the other pairings was examined in our cell-based experiments.

### Discovery of covariation pairs between 5′UTR_243_ nucleotide and NS2/3 amino acid residues

**Figure 2:**
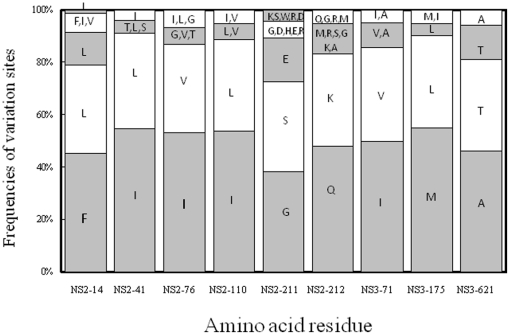
Frequencies of variation sites assessed from Weka. The covariation sites were predicted at the 5′UTR_243_ nucleotide position and their corresponding amino acid sites in HCV NS2 or NS3. Co-variation sites between the 5′UTR_243_G (grey) and 5′UTR_243_A (white) with the corresponding amino acid residues (single-letter code) are shown in accumulative percentage. A frequency >10% for an amino acid residue is presented individually. Possible NS2 co-evolving sites include the 14^th^, 41^st^, 76^th^, 110^th^, 211^th^ and 212^th^ residues; possible NS3 sites include the 71^st^ 175^th^ and 621^st^ residues.

The data mining results showed a strong covariation relationship of the 243^rd^ nucleotide of 5′UTR to the 14^th^, 41^st^, 76^th^, 110^th^, 211^th^ and 212^th^ residues of NS2 and to the 71^st^, 175^th^ and 621^st^ residues of NS3 (Fig. 2). Notably, the 5′UTR_243_G was frequently associated with NS2-14F, -41I, -76I, -110I, -211G, -212Q, NS3-71I, -175M and 621A, while the 5′UTR_243_A was associated with NS2-14L, -41L, -76V, -110L, -211S, -212K, NS3-71V, -175L and 621T. Neither C nor T was present at the 243^rd^ nucleotide of the 5′UTR. Simultaneous change of both sites of a covariant pair, as opposed to the change of a single site of a covariant pair, has been hypothesized to be better for HCV replication. With this in mind, the HCV replication consequences of the observed covariations were tested in a cell-based system, as detailed in the following.

### Cell-based functional analysis - Evaluating HCV replication efficiency by mutation of coordinated variations between 5′UTR_243_ nucleotide and NS2/NS3 amino acid residues by using NS2-3′ replicon

**Figure 3:**
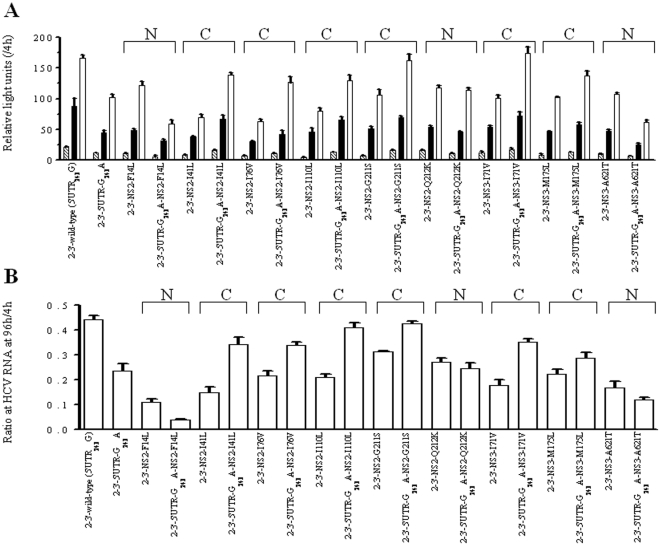
Transient-replication assay of NS2-3′ replicons. The replicons carried either wild-type (5′UTR_243_G) or 5′UTR-G_243_A and 9 pairs of variants carrying specific amino acid residues as indicated at the NS2 or NS3 regions. Huh-7 cells were transfected with 2.5 µg of replicon RNA. (A) Luciferase activity was determined in cell lysates at 48h (stripe), 72 h (black) and 96 h (white) posttransfection. Data were normalized by the values at 4 h posttransfection and expressed as mean ± SD (n = 4). (B) HCV-RNA was quantified by real-time PCR at 96 h posttranfection. Data were normalized by the values at 4 h posttransfection and expressed as mean ± SD (n = 3). Note that replication efficiency of replicons carrying amino acid variants was compensated by the presence of 5′UTR-G_243_A, indicated as compensatory pair (C) and otherwise as non-compensatory pair (N).

**Figure 4:**
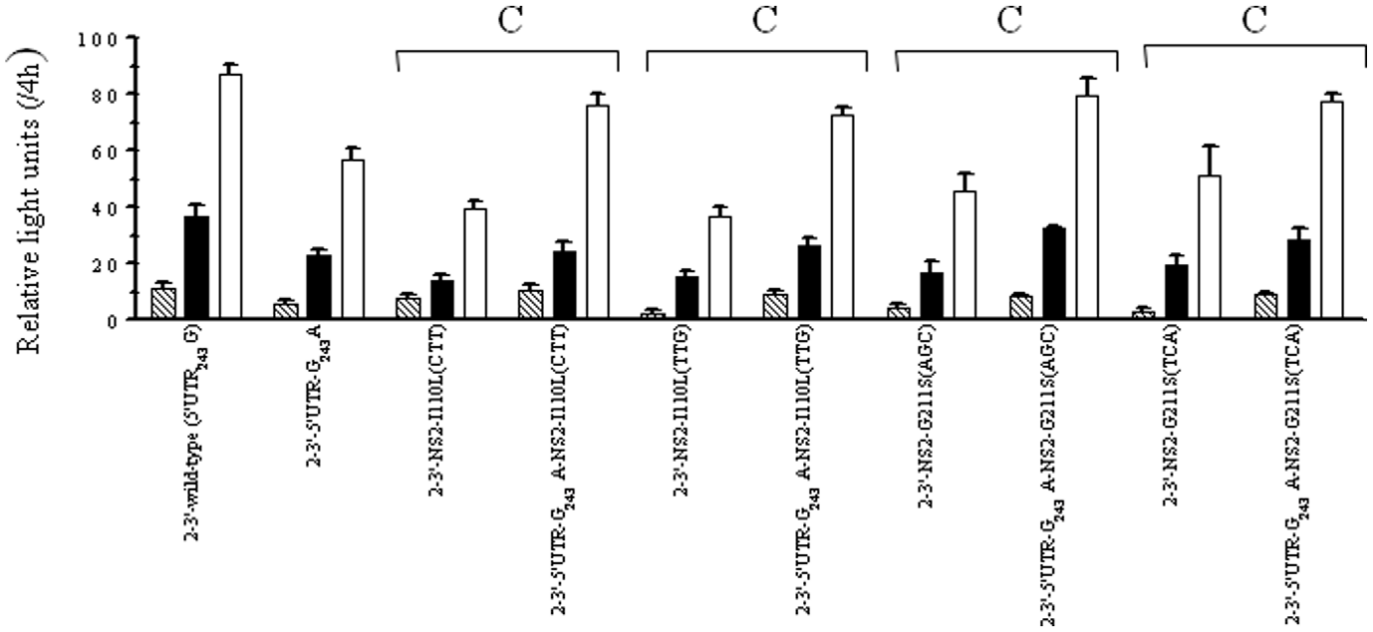
Transient-replication assay of NS2-3′ replicons. The replicons carried different types of codon usage for NS2-I110L (CTT and TTG) and NS2-G211S (AGC and TCA). Huh-7 cells were transfected with 2.5 µg of replicon RNA. Resulting luciferase activities were determined from equal amounts of cell lysate harvested at 48 h (stripe), 72 h (black) and 96 h (white) posttransfection. Data were normalized by luciferase activity measured at 4 h posttransfection and expressed as mean ± SD (n = 4). Compensatory pairs (C) are indicated as described in Fig. 3.

Site-specific mutations matching various of the observed covariations were introduced in order to analyze their effects on the replication efficiency using a transient-replication assay. We constructed 9 pairs of variants in the context of the wild-type NS2-3′ replicon (5′UTR_243_G), each consisting of a single amino acid substitution at the NS2 or NS3 region and double substitutions in combination with 5′UTR-G_243_A and the corresponding amino acid (Figs. 3A and B). Based on the normalized luciferase activities at 3 consecutive time points, the transient luciferase assays indicated that the 9 single amino acid variants decreased replication efficiency in the presence of 5′UTR_243_G, but replication efficiency could be rescued when any single variant of NS2-I41L, NS2-I76V, NS2-I110L, NS2-G211S, NS3-I71V and NS3-M175L was combined with 5′UTR-G_243_A. On the contrary, the 5′UTR-G_243_A could not compensate the NS2-F14L, NS2-Q212K and NS3-A621T variants. Furthermore, different types of codon usage were introduced for NS2-I110L (CTT and TTG) and NS2-G211S (AGC and TCA), yielding comparable compensatory effects and indicating that differences of codon usage at the nucleotide level may not be a concern (Fig. 4). These results together suggest that the covariation of 5′UTR-G_243_A with the NS2 and NS3 proteins was most likely due to amino acid substitution, but this was not the case for the specific nucleotide sequences.

### Dependence of NS2 protein in functional coordinated variations between 5′UTR_243_ and NS3

**Figure 5:**
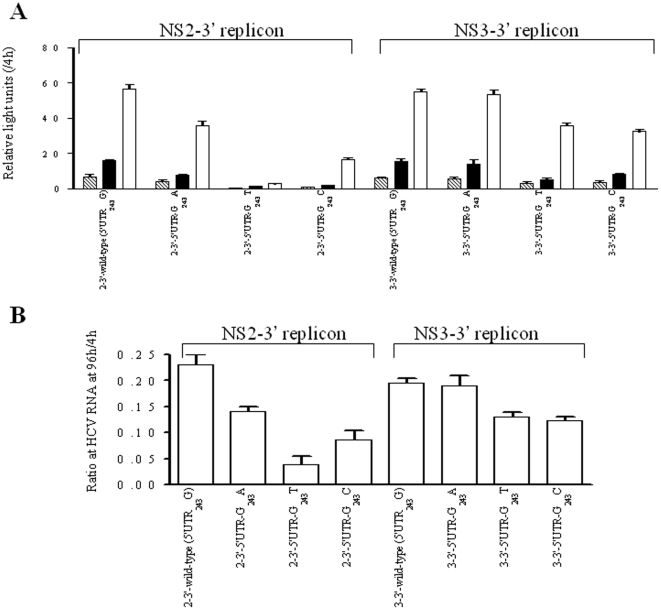
Transient-replication assay of the NS2-3′ and NS3-3′ replicons. The replicons each carried wild-type (5′UTR_243_G), 5′UTR-G_243_A, 5′UTR-G_243_T and 5′UTR-G_243_C. Huh-7 cells were transfected with 2.5 µg of replicon RNA. (A) Luciferase activity was determined in cell lysate at 48 h (stripe), 72 h (black) and 96h (white) posttransfection. Data were normalized by the values at 4 h posttransfection, expressed as mean ± SD (n = 4). (B) HCV-RNA was quantified by real-time PCR at 96 h posttranfection. Data were normalized by the values at 4 h posttransfection, expressed as mean ± SD (n = 3).

**Figure 6:**
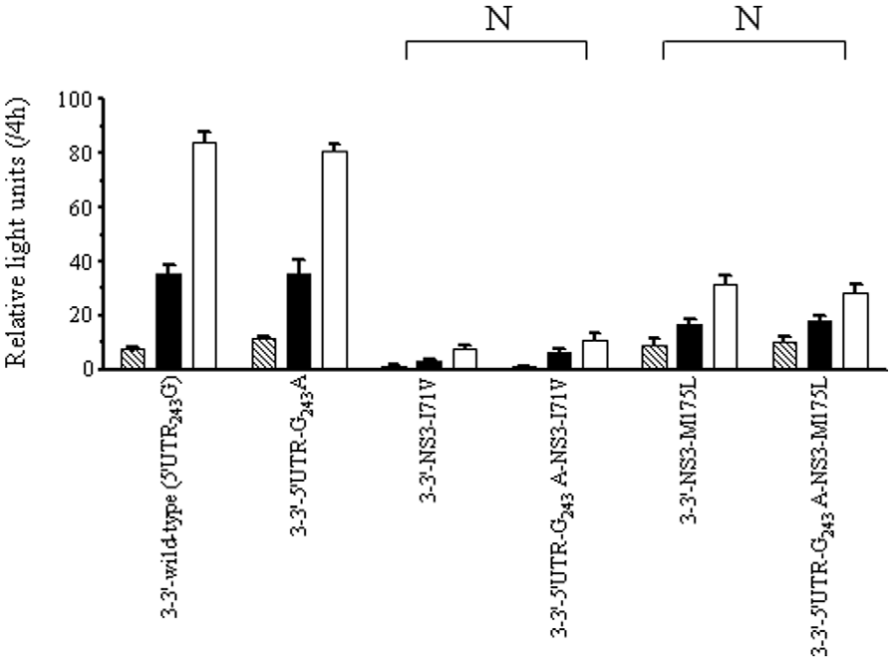
Transient-replication assay of NS3-3′ replicons. The replicons carried either wild-type (5′UTR_243_G) or 5′UTR-G_243_A and 2 pairs of variants carrying NS3-I71V and NS3-M175L. Huh-7 cells were transfected with 2.5 µg of replicon RNA. Luciferase activity was determined in cell lysate at 48 h (strip), 72 h (black) and 96 h (white) posttransfection. Data were normalized by the values at 4 h posttransfection, expressed as mean ± SD (n = 4). Non-compensatory pairs (N) are indicated as described in Fig. 3.

The effects of nucleotide substitution at the 5′UTR_243_ site with regard to HCV replication were compared for the NS2-3′ or NS3-3′ replicon backbone contexts. Compared to 5′UTR_243_G in the NS2-3′ replicon context, 5′UTR-G_243_A showed a moderate decline in replication efficiency, whereas 5′UTR-G_243_T and 5′UTR-G_243_C showed a profound decline (Figs. 5A and B). However, 5′UTR-G_243_A, 5′UTR-G_243_T and 5′UTR-G_243_C showed no or little influence on replication efficiency in the context of the NS3-3′ replicon (Figs. 5A and B). These results indicate that the NS2 protein may be of great importance in replicative modulation mediated by HCV 5′UTR_243_. Further, the NS3-I71V and NS3-M175L variants impaired HCV replication to similar levels in the NS3-3′ replicons carrying either 5′UTR_243_G or 5′UTR-G_243_A (Fig. 6). Because 5′UTR-G_243_A could only compensate NS3-I71V and NS3-M175L in the presence of NS2 (Fig. 3), the results suggest that these functional coordinated variations between 5′UTR_243_ and NS3 depend on the NS2 protein.

### Modulation of HCV replication efficiency by exogenously expressed NS2 proteins using NS3-3′ replicon

**Figure 7:**
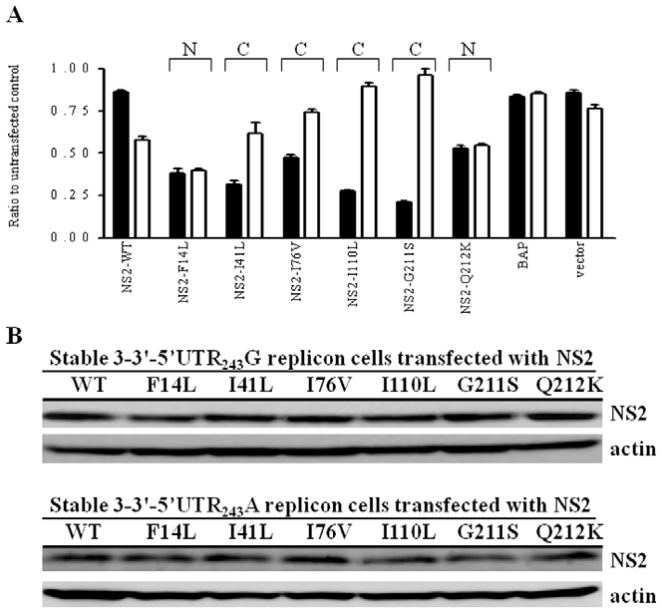
Modulation of HCV replication by exogenously expressed NS2 protein. (A) Three micrograms of each wild-type or variant NS2-flag expression plasmids were transfected into stable NS3-3′-Feo-5′UTR_243_G (black bar) or NS3-3′-Feo-5′UTR_243_A (white bar) replicon cells. Cell lysate was harvested at 48 h posttransfection and luciferase activity was determined. Data were calculated as the ratio to the corresponding untransfected control, expressed as mean ± SD (n = 4). (B) Fifty micrograms of total protein from each cell lysate were separated by 12% sodium dodecyl sulfate-polyacrylamide gel electrophoresis and immunostained with antibodies recognizing the flag epitope. Compensatory pairs (C) and non-compensatory pairs (N) are indicated as in Fig. 3.

Next, we addressed the question of whether the NS2 protein variants expressed exogenously could modulate HCV replication activity of the HCV NS3-3′replicon in the presence of 5′UTR_243_G or 5′UTR_243_A. Wild-type and variant forms of the NS2-flag fusion protein were transfected into stable NS3-3′-Feo-5′UTR_243_G or NS3-3′-Feo-5′UTR_243_A replicon cells, after which the lysate luciferase activities were analyzed (Fig. 7). The results showed that the wild-type NS2 reduced by ∼10% the replication activity of the NS3-3′-Feo-5′UTR_243_G replicon and by ∼40% the replication activity of the NS3-3′-Feo-5′UTR_243_A replicon. As compared to the wild-type NS2, the 6 individual variant NS2 proteins substantially reduced the replication activity of the NS3-3′-Feo-5′UTR_243_G replicon. However, the replication efficiencies reduced by NS2-I41L, NS2-I76V, NS2-I110L and NS2-G211S could be rescued in the NS3-3′-Feo-5′UTR_243_A replicon, but not those reduced by NS2-F14L, NS2-Q212K (Fig. 7A). The NS2-flag proteins were immunostained by western blot analysis, exhibiting at levels comparable to the 7 proteins, i.e. the wild-type and the six variants (Fig. 7B). Thus, the results of the exogenously expressed wild-type and the variant NS2 agreed with the experiments based on NS2 expressed as replicon NS proteins, indicating that the compensatory effects did not depend on whether the NS2 proteins were expressed as a separate protein or in a polyprotein.

### Regulation of HCV replication by 5′UTR-mediated NS2 binding to HCV RNA

**Figure 8:**
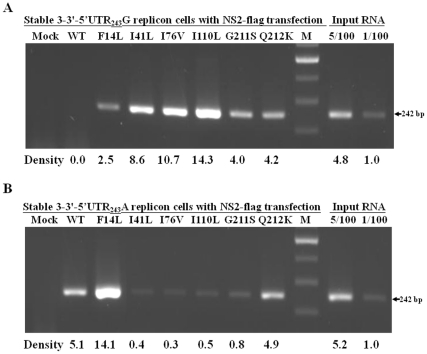
Immunoprecipitation of RNA-protein assay using anti-flag antibody recognized crosslinked HCV NS2-flag protein and replicon RNA. Stable NS3-3′-Feo-5′UTR_243_G (A) or NS3-3′-Feo-5′UTR_243_A (B) replicon cells transfected with each of the wild-type and variant NS2-flag expression plasmids were harvested for formaldehyde-mediated crosslinking. After immunoprecipitation with anti-flag antibody, purified RNA samples were analyzed by reverse transcription-PCR using a primer pair spanning 242 bases from the HCV 5′UTR region. Total RNA samples from mock transfected replicon cells were analyzed by reverse transcription-PCR in parallel. Band intensity quantification was performed by AlphaImage 2200 software and a representative experimental run is shown (Lab Recyclers, MD).

To study the RNA/protein interactions, stable NS3-3′-Feo-5′UTR_243_G or NS3-3′-Feo-5′UTR_243_A replicon cells transfected with each of the wild-type and variant NS2-flag expression plasmids were crosslinked at 48 hr after transfection. Immunoprecipitation of the RNA-protein complexes was performed using anti-flag antibody to specifically recognize HCV NS2-flag protein. Bound RNA samples were then detected by reverse transcription-PCR using HCV-specific primers at the 5′UTR region. The results showed that HCV-specific 242-bp product could be detected in the NS2-F14L, NS2-I41L, NS2-I76V, NS2-I110L, NS2-G211S and NS2-Q212K replicon cells that expressed NS3-3′-Feo-5′UTR_243_G, but not in the wild-type NS2 transfectant (Fig. 8A). On the other hand, the HCV-specific 242-bp product was detected in the wild-type NS2, NS2-F14L and NS2-Q212K replicon cells that expressed NS3-3′-Feo-5′UTRG_243_A, but could only barely be detected in those transfected with NS2-I41L, NS2-I76V, NS2-I110L and NS2-G211S (Fig. 8B). The stronger ability of NS2 to bind to the HCV-RNA of the NS2 variants relative to the wild-type HCV-RNA correlated with decreasing HCV replication in the 5′UTR_243_G replicon cells, as shown in Figs. 3 and 7. As the NS2/HCV-RNA binding abilities of the NS2-I41L, NS2-I76V, NS2-I110L and NS2-G211S variants weakened in the 5′UTR_243_A replicon cells, HCV replication levels were restored. Together, these data indicate that 5′UTR-mediated NS2 binding to HCV RNA can regulate HCV replication.

### Genotype-specific covariation patterns between 5′UTR_243_ nucleotide and specific NS2/NS3 amino acid residues

Analysis of the frequencies of the co-evolutionary sites in the 217 sampled HCV genome found distinct dominant sequences between the 1b and the non-1b genotype groups (Table S3). To confirm the genotype-specific covariation patterns, 381 additional full-length HCV genome sequences in an updated Los Alamos HCV sequence database were downloaded on May 19, 2009, making a total of 598 (217 in the Nov. 30, 2006 download + 381 in the May 19, 2009 download  =  598 total) analyzed nucleotide sequences. The combined results showed that G-Ile-Ile-Ile-Gly-Ile-Met was predominant (appearing in 53.8% of genotype 1b but 0.0% of genotype non-1b) for genotype-1b, while A-Leu-Val-Leu-Ser-Val-Leu was predominant (appearing in 79.1–100.0% of genotype non-1b but 0.0% of genotype 1b) in genotypes 1a, 2a and 2b (Table 1). These results confirmed that the initial data mining dataset for the nucleotide and amino acid residues found in 5′UTR, NS2 and NS3 co-varied in a genotype-specific manner.

**Table 1:** Comparison of the predominant haplotypes of 5′UTR_243_, NS2-41, -76, -110, -211, NS3-71 and -175 among the HCV genotypes.

HCV genotype	Number of full-length HCV sequences (n = 598)	Haplotype of 5′UTR_243_, NS2-41, -76, -110, -211, NS3-71 and -175	
		G-Ile-Ile-Ile-Gly-Ile-Met	A-Leu-Val-Leu-Ser-Val-Leu
1a	218 (100%)^a^	0 (0.0%)	218 (100.0%)
1b	327 (100%)	176 (53.8%)	0 (0.0%)
2a	29 (100%)	0 (0.0%)	29 (100.0%)
2b	24 (100%)	0 (0.0%)	19 (79.1%)

a Number (percentage).

## Discussion

Data mining involves finding patterns or rules in large data sets. Such patterns can be used to make predictions or form the basis of hypotheses for future experiments [26]-[28]. Data mining is being integrated into bioinformatic research [29]. In the present study, data mining methodology found previously unnoticed nonrandom covariance between HCV 5′UTR with NS2 and NS3 proteins from a large HCV genomic database built from patient samples. This nonrandom association was experimentally verified to be of functional significance to viral replication by use of a cell-based HCV replicon system.

Protein residue covariation may suggest physical and/or functional constraints of paired amino acid positions [30]. As shown in previous studies, the covarying amino acid residues in the 10 HCV proteins display a scale-free network where central amino acid substitutions connect to many other sites [18], [31]. Data mining analysis in the present study has revealed that coordinated variations occur between the untranslated 5′UTR-RNA elements and the amino acid residues of the NS2 and NS3 proteins. UTRs are traditionally thought to have no influence on protein coding sequences. Accordingly, the data mining results of this study indicating coordinated variations between the 5′UTR-RNA element and the NS2/NS3 proteins were surprising. Importantly, the computational results were confirmed by cell-based experiments using replicon replication and RNA-protein interaction assay to have significant effect on viral replication. Therefore, this study demonstrates a functionally significant pattern of linkage disequilibrium involving a non-coding nucleotide (5′UTR_243_) and the amino acid residues (4 NS2 sites and 2 NS3 sites) in the HCV genome. The results suggest mutual communication in *trans* between HCV 5′UTR-RNA and individual NS2 proteins, or a combination of NS2 and NS3 proteins, by a mechanism that possibly involves direct binding or interaction with a common partner from either the HCV or host factors such as cellular protein or RNA. Strong binding of the NS2 proteins to the HCV 5′UTR-RNA appears to diminish HCV replication, whereas weak binding correlates with restoration of HCV replicative efficiency.

In cell-based systems, HCV NS2 is not an indispensable component for replication because HCV subgenomic replicon RNA allows replication in the absence of NS2 [32], [33]. However, the NS2 protein may modulate IRES-dependent translation, NS3 kinetics and/or NS5B replication, thus affecting HCV synthesis of both viral RNA and proteins [34], [35], and also may mediate HCV assembly and release [36], [37]. It has been reported that NS2 sequences differ between nonresponder and relapser groups in HCV patients receiving antiviral therapy, with clinical relevance [38]. According to NS2 topology [12], the 14^th^, 41^st^ and 76^th^ residues are located at the first, the second and the third transmembrane domains, respectively. The present study suggests a novel regulatory mechanism involving NS2, whereby NS2 with a high binding affinity for 5′UTR sequences may result in reduced HCV RNA flexibility, which in turn may compromise HCV RNA conformational rearrangement and/or the joining of other essential factors, resulting in less efficient HCV replication.

HCV 5′UTR_243_ is located at a non-Watson-Crick base pair position between the IRES IIIc and IIId domains of the positive strand [5], [39], [40] and at the IIIc'domain of the replicative strand [41]. Both the positive- and negative-stranded domains of this non-coding region function as host protein binding sites which regulate translation and replication [42], [43]. In previous studies using either rabbit reticulocyte or hepatic lysates *in vitro*, 5′UTR_243_A and 5′UTR_243_G had the same IRES translation activities [40], [44], [45]. Furthermore, a G-to-A change at 5′UTR_243_ exhibited preferential translation functions in lymphoblastoid cell lines and primary dentritic cells [45]–[47], suggesting that 5′UTR_243_ might be a cell-specific determinant in viral RNA translation. The present study revealed that changes in 5′UTR_243_ alter HCV replication, with G, A, C and T displayed in order of decreasing activity with the NS2-3′ replicon assay. These results agree with G and A, but not with C and T at 5′UTR_243_ in the HCV from patient samples, i.e. the HCV sequences in patient samples show G and A, but no C and T at the 5′UTR_243_ site. In addition, our results indicate that HCV 5′UTR_243_ may mutate in a genotype-specific covariant manner. The dominant haplotype of 5′UTR_243_-NS2-41-76-110-211-NS3-71-175 differed among HCV 1b and non-1b genotypes. This variance relationship could only be seen in a population consisting of 1b and non-1b genotypes, but not in the 1b or non-1b subpopulations alone. The distinct haplotype patterns suggest that the genotype-1b may split from the non-1b genotypes where fitness epistasis causes fixation of beneficial polymorphisms within a genotypic subpopulation. The genotype-1b haplotype was G-Ile-Ile-Ile-Gly-Ile-Met for 5′UTR_243_, NS2-41, -76, -110, -211, NS3-71 and -175; that of the non-1b haplotype was A-Leu-Val-Leu-Ser-Val-Leu. It should be noted that genotype-1b had 4 Ile residues but that genotype-non-1b had none. Genotype-non-1b had 3 Leu residues and 2 Val residues while genotype-1b had none, suggesting that covarying substitutions differ but that the physicochemical properties in these hydrophobic residues may be conserved between genotypes 1b and non-1b. These sites may be of significance in determining HCV functional changes during genome evolution.

Adaptive mutations at the NS regions in cell-culture based systems have been shown in prior work [25], [48]. A recent study further identified an adapted Gly-to-Arg mutation at the 28^th^ residue of NS2 in a chimpanzee-infected JFH-1 strain [49]. This present study reports coevoluationary sites of the NS2 and NS3 proteins in humans. Covariance of these sites during divergent genome evolution is assumed to be advantageous to HCV replication *in vivo*. It is most likely that the primary mutations appear at residues of the NS2 and NS3 proteins, subsequently exerting structural-dynamic pressure that induces a conformational change of 5′UTR_243_, which is located in the most conserved region of the HCV genome.

In conclusion, the presented data mining analysis of HCV genome sequences has indicated by both computational methodology and by cell-based HCV replicon assay that 5′UTR_243_ and specific residues of the NS2 and NS3 proteins are involved in a previously unnoticed nucleotide and amino acid covariation, which may be associated with genome evolution which contributes to functional regulation of HCV replication. These results further support the premise that data mining methodology is an effective tool for finding useful patterns in the increasingly large database of contemporary virus research.

## Materials and Methods

### Data mining analysis

The employed data mining analysis involved the following steps: i) full-length HCV genome sequences were downloaded from the Los Alamos HCV database [24]; ii) the nucleotide segments of the UTRs and the amino acid segments of the NS proteins were retrieved and combined, creating 6 new binary sequence components including 5′UTR-NS2, 5′UTR-NS3, 5′UTR-NS5B, NS2-3′UTR, NS3-3′UTR and NS5B-3′UTR (Fig. 1); iii) multiple sequence alignments were constructed by the CLUSTAL W software program [50] and confirmed by visual inspection; iv) 100% conserved columns in multiple sequence alignment were eliminated by GeneDoc to avoid false covariation signals due to site conservation; v) the covariation relationships of the remaining individual sites were identified by association rule mining based on the Apriori algorithm using Weka software [23]. A format transformation system was used to transform the output of GeneDoc into ARFF format which is readable as Weka input. The Apriori algorithm satisfies 2 parameters: support (also known as coverage, proportion of instances that contain a particular code) and confidence (also known as accuracy, proportion of instances that it predicts correctly) to find the best association rules. In order to capture novel covariations from highly polymorphic sites, the support threshold was set at 0.33. In order to identify strong associations, the minimum confidence threshold was set at 1.0, which indicates that the identified rule is present in 100% of the sequences.

### Cell cultures

Cell monolayers of cloned human Huh7 hepatoma cell line were grown in Dulbecco's modified Eagle medium (HyClone, USA) supplemented with 10% heat-inactivated fetal bovine serum and 1% penicillin/streptomycin at 37°C in a 5% CO_2_ atmosphere.

### Plasmid construction

The HCV replicon constructs pFK-i341-PI-Luc/NS2-3′/ET (pNS2-3′ replicon) and pFK-i341-PI-Luc/NS3-3′/ET (pNS3-3′ replicon) for transient replication were kindly provided by Professor R. Bartenschlager [25]. These constructs possessed the luciferase reporter gene and the NS gene frame starting from either NS2 or NS3 to NS5B and were used as backbone constructs to generate co-variation mutants. G-to-A mutation in the 5′UTR and its paired NS2 or NS3 covariant mutations were introduced into the backbone constructs by site-directed mutagenesis using the QuickChange XL Site-Directed Mutagenesis kit (Stratagene, USA). The mutation sites generated in the NS2 region were F14L, I41L, I76V, I110L, G211S or Q212K; the mutation sites generated in the NS3 region were I71V, M175L or A621T. The oligonucleotides used for construction of replicon variants are listed in Table S4. To facilitate stable selection of covariant mutants, a gene fragment composed of fused firefly luciferase and neomycin phosphotransferase genes (Feo) was used to replace the firefly luciferase gene. The coding regions of the wild-type and the variant NS2 were PCR amplified from the corresponding replicon templates and subcloned into a p3XFLAG-CMV-14 expression vector (Sigma-Aldrich, Germany). DNA sequencing was used to verify the exact site-specific substitutions. No non-target sequence changes were introduced.

### *In vitro* transcription, electroporation and transient replication assay

*In vitro* transcripts were prepared and transfection by electroporation was carried out as described previously [25], with slight modifications. Briefly, the replicon plasmids were prepared with a midi-plasmid extraction kit (Qiagen, USA), linearized with *Ase*I and *Sca*I (New England Biolabs, USA), extracted with phenol and chloroform, precipitated with ethanol and dissolved with nuclease-free water. T7 promoter-driven *in vitro* transcription was performed with purified linearized replicon DNA using MEGAscript T7 kit (Ambion, USA) at 37°C for 2 hours. The DNA templates were digested by adding RNase-free DNase. After purification by RNeasy MinElute Cleanup kit (Qiagen), the replicon RNA was precipitated with alcohol and dissolved with nuclease-free water. RNA quantity and purity was determined by 260nm/280nm optical density measurements and agarose gel electrophoresis.

For electroporation, monolayered Huh7 cells were trypsinized and resuspended at a concentration of 5×10^6^ cells per mL in cytomix buffer [51] containing 2 mM of ATP and 5 mM of glutathione. Four-hundred microliters of the suspended cells were mixed with 2.5 µg of replicon RNA and 5 µg of total RNA from the Huh7 cells as a carrier. The cell mixture was transferred to a 4-mm cuvette and electroporated at 1300 V for 99 µsec using electroporation equipment (ECM 830, BTX Harvard Apparatus, USA). After incubation at room temperature for 10 minutes, the cells were seeded into a 6-well and harvested at given time points after transfection.

For transient replication assay, the cells were washed with 1x PBS and lysed with 1x passive lysis buffer (Promega, USA), 120 µL per well. Then, 20 µL of supernatant was mixed with 100 µL of Luciferase assay reagent (QuantiLum Recombinant Luciferase kit, Promega) and measured in a luminometer (Lumat LB9506, Berthold Technologies, Germany). The values of luciferase activity in the cell lysates harvested at 4 h posttransfection were used to normalize the transfection efficiency.

### Quantitation of HCV RNA by real-time PCR

Total RNA was isolated from cells with a single-step method modified from the acid guanidinium–thiocyanate–phenol–chloroform extraction procedure with REzolTM C&T reagent (Protech Technology, Taiwan). Intracellular HCV-RNA titers were measured quantitatively by reverse transcription coupled to real-time PCR assay using a LightCycler instrument (Roche, Germany) yielding a dynamic range from 800 to 100 million copies/mL [52].

### Stable HCV replicon cells

At 48 h posttransfection, the Huh7 cells transfected with plasmids carrying Feo gene were selected by 500 µg/mL G418 for approximately 1 month to obtain stable NS3-3′-Feo-5′UTR_243_G and NS3-3′-Feo-5′UTR_243_A replicon cells.

### Antibodies and Western blot analysis

Monoclonal antibodies specific to flag tag (clone F1804) and actin (clone MAB1501) were purchased from Sigma-Aldrich (USA) and Chemicon (USA), respectively. Cell lysates were electrophoresed on sodium dodecyl sulfate-polyacrylamide gel electrophoresis and transferred to a polyvinylidene fluoride membrane. After blocking, the membrane was incubated with specific primary antibody, washed with 0.05% phosphate buffer saline-Tween 20, reacted with horseradish peroxidase-conjugated secondary antibody and developed with Western Lighting (Perkin-Elmer, USA).

### Immunoprecipitation of RNA-protein assay

Immunoprecipitation of RNA-protein complexes was modified from the method in [53]. Wild-type and variant NS2 expression plasmids were transfected into stable HCV replicon cells by lipofectamin 2000 (Invitrogen, USA). At 48 h posttransfection, 1×10^6^ cells were harvested by trypsinization and then crosslinked by 1% formaldehyde in phosphate buffer saline at room temperature for 30 min, followed by a quench solution (0.25 M glycine in phosphate buffer saline). The cells were then resuspended in RIPA buffer (50 mM Tris-HCl, pH 7.5, 1% Nonidet P-40, 0.5% sodium deoxycholate, 0.05% sodium dodecyl sulfate, 1 mM EDTA, 150 mM NaCl) containing a cocktail of protease inhibitors (Roche) and RNase inhibitor (Takara, Japan) and lysed by 3 freeze–thaw cycles. After centrifugation at 12000 rpm for 15 min to remove any insoluble materials, the clarified supernatant was collected, pre-cleared with protein G beads not coupled with ligand but accompanied by yeast tRNA as a nonspecific competitor, and then incubated with protein G beads coupled with anti-flag monoclonal antibody at room temperature for 90 min. The complex was washed with RIPA buffer, resuspended in a solution of 50 mM Tris-HCl, pH 7.0, 5 mM EDTA, 10 mM dithiothreitol and 1% sodium dodecyl sulfate, then incubated at 70°C for 45 min to reverse the crosslinking. The immunoprecipitated RNA was analyzed by reverse transcription-PCR. Briefly, the RNA was extracted with Rezol C&T (Protech) reagent, reversely transcribed into cDNA with Moloney murine leukemia virus RT (Promega) and PCR amplified for 35 cycles with a primer pair (sense primer: 5′-ACTCCACCATAGATCACTCC-3′ and antisense primer: 5′-AACACTACTCGGCTAGCAGT-3′) spanning 242 bases from the HCV 5′UTR region.

## Supporting Information

Figure S1 **Neighbor-joining phylogenetic tree of the HCV sequences.** The sequences were downloaded from the Los Alamos HCV database on Nov. 30, 2006. 217 full-length HCV genome sequences were aligned using CLUSTAL software and phylogenetically analyzed by the neighbor-joining method using the molecular evolutionary genetics analysis (MEGA) program. The constructed phylogenetic tree includes 19 sequences for 1a genotype (▪), 127 for 1b (□), 4 for 1c (▴), 22 for 2a (•), 23 for 2b (○), 1 each for genotypes 2c and 2k (▾), 4 each for 3a and 3b and 1 for 3k (▽), 1 for 4a (△), 2 for 5a (⧫), 2 each for 6a and 6k and 1 each for 6b, 6d, 6g and 6h (◊).
(TIF)

Table S1 **Genotypic distribution and accession numbers of the 217 full-length HCV genome sequences.**
(DOCX)

Table S2 **Summary of the unique association rules.**
(DOCX)

Table S3 **The frequencies (%) of co-evolutionary sites in the sampled HCV genome sequences.**
(DOCX)

Table S4 **Oligonucleotides used for construction of replicon variants.**
(DOCX)
