# Supplementary figures and images for: Novel Nucleotide and Amino Acid Covariation between the 5′UTR and the NS2/NS3 Proteins of Hepatitis C Virus: Bioinformatic and Functional Analyses
Source: PLoS One. 2011 Sep 28;6(9):e25530. doi: 10.1371/journal.pone.0025530 (PMC3182228; doi:10.1371/journal.pone.0025530)

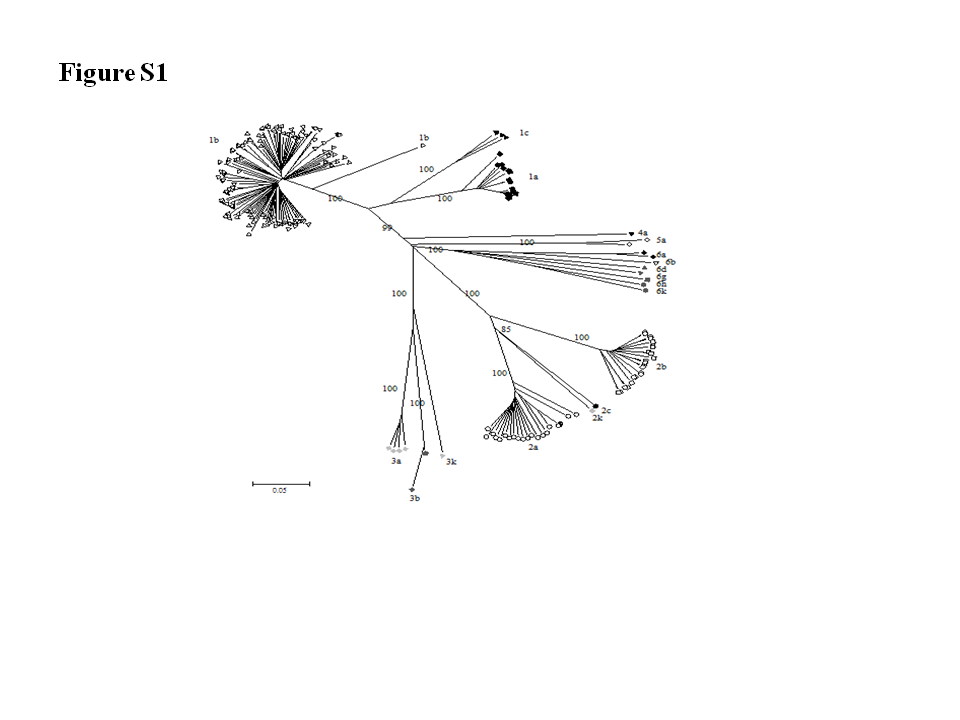

Supplement: Figure S1 — Neighbor-joining phylogenetic tree of the HCV sequences. The sequences were downloaded from the Los Alamos HCV database on Nov. 30, 2006. 217 full-length HCV genome sequences were aligned using CLUSTAL software and phylogenetically analyzed by the neighbor-joining method using the molecular evolutionary genetics analysis (MEGA) program. The constructed phylogenetic tree includes 19 sequences for 1a genotype (▪), 127 for 1b (□), 4 for 1c (▴), 22 for 2a (•), 23 for 2b (○), 1 each for genotypes 2c and 2k (▾), 4 each for 3a and 3b and 1 for 3k (▽), 1 for 4a (△), 2 for 5a (⧫), 2 each for 6a and 6k and 1 each for 6b, 6d, 6g and 6h (◊). (TIF) [file pone.0025530.s001.tif]
