# Supplementary material for: Novel Nucleotide and Amino Acid Covariation between the 5′UTR and the NS2/NS3 Proteins of Hepatitis C Virus: Bioinformatic and Functional Analyses
Source: PLoS One. 2011 Sep 28;6(9):e25530. doi: 10.1371/journal.pone.0025530 (PMC3182228; doi:10.1371/journal.pone.0025530)
Supplement: Table S1 — Genotypic distribution and accession numbers of the 217 full-length HCV genome sequences. (DOCX) [file pone.0025530.s002.docx]

**Table S1**. **Genotypic distribution and accession numbers of the 217 full-length HCV genome sequences.**

| Genotype | Accession numbers |
| --- | --- |
| 1a (n=19) | AF009606, AF011751, AF011752, AF011753, AF271632, AF290978  AF511948, AF511949, AF511950, AJ278830, AX100563, AX663428  D10749, E08263, E08264, E66593, M62321, M67463, NC_004102 |
| 1b (n=127) | AB016785, AB049087, AB049088, AB049089, AB049090, AB049091  AB049092, AB049093, AB049094, AB049095, AB049096, AB049097  AB049098, AB049099, AB049100, AB049101, AB080299, AB191333  AF054247, AF054248, AF054249, AF054250, AF139594, AF165045  AF165046, AF165047, AF165048, AF165049, AF165050, AF165051  AF165052, AF165053, AF165054, AF165055, AF165056, AF165057  AF165058, AF165059, AF165060, AF165061, AF165062, AF165063  AF165064, AF176573, AF207752, AF207753, AF207754, AF207755  AF207756, AF207757, AF207758, AF207759, AF207760, AF207761  AF207762, AF207763, AF207764, AF207765, AF207766, AF207767  AF207768, AF207769, AF207770, AF207771, AF207772, AF207773  AF207774, AF208024, AF333324, AF356827, AF483269, AJ000009  AJ132996, AJ132997, AJ238799, AJ238800, AX036252, AX036258  AX036260, AX036262, AX739971, AY045702, AY460204, AY587016  AY587844, CQ819761, D10750, D10934, D11168, D11355, D13558  D14484, D30613, D45172, D50480, D50481, D50482, D50483, D50484, D50485, D63857, D85516, D89815, D89872, D90208, E03766, E04420, E05027, E06261, E06457, E07579, E08399, E08461, E09631, E10035, L02836, M58335, M84754, M96362, S62220, U01214, U16362, U45476  U89019, X61596, AY587845, DQ071885 |
| 1c (n=4) | AY651061, AY051292, D14853, E08443 |
| 2a (n=22) | AB047639, AB047640, AB047641, AB047642, AB047643, AB047644  AB047645, AF169002, AF169003, AF169004, AF169005, AF177036  AF238481, AF238482, AF238483, AF238484, AF238485, AX057086  AX057317, AX057395, AY746460, D00944 |
| 2b (n=23) | AB030907, AF238486, AY232730, AY232731, AY232732, AY232733  AY232734, AY232735, AY232736, AY232737, AY232738, AY232739  AY232740, AY232741, AY232742, AY232743, AY232744, AY232745  AY232746, AY232747, AY232748, AY232749, D10988 |
| 2c (n=1) | D50409 |
| 2k (n=1) | AB031663 |
| 3a (n=4) | AF046866, D17763, D28917, X76918 |
| 3b (n=4) | D49374, E10839, E10840, E10841 |
| 3k (n=1) | D63821 |
| 4a (n=1) | Y11604 |
| 5a (n=2) | Y13184, AF064490 |
| 6a (n=2) | AY859526, Y12083 |
| 6b (n=1) | D84262 |
| 6d (n=1) | D84263 |
| 6g (n=1) | D63822 |
| 6h (n=1) | D84265 |
| 6k (n=2) | AY878650, D84264 |
