# Supplementary material for: Novel Nucleotide and Amino Acid Covariation between the 5′UTR and the NS2/NS3 Proteins of Hepatitis C Virus: Bioinformatic and Functional Analyses
Source: PLoS One. 2011 Sep 28;6(9):e25530. doi: 10.1371/journal.pone.0025530 (PMC3182228; doi:10.1371/journal.pone.0025530)
Supplement: Table S2 — Summary of the unique association rules. (DOCX) [file pone.0025530.s003.docx]

**Table S2.** **Summary of the unique association rules.**

|  | Nucleotide | Amino acid residue |
| --- | --- | --- |
| All genotypes  (no. of rules = 12) | 5'UTR_204_ | NS3-71, NS3-175, NS3-621 |
|  | 5'UTR_243_ | NS2-14, NS2-41, NS2-76, NS2-110, NS2-211, NS2-212 |
|  | 5'UTR_243_ | NS3-71, NS3-175, NS3-621 |
|  |  |  |
| Genotype 1a  (no. of rules = 11) | 5'UTR_204_ | NS5B-130, NS5B-444, NS5B-544 |
|  | 3'UTR_32_ | NS2-169, NS2-199 |
|  | 3'UTR_32_ | NS5B-117, NS5B-444 |
|  | 3'UTR_88_ | NS2-169, NS2-199 |
|  | 3'UTR_117_ | NS2-169, NS2-199 |
|  |  |  |
| Genotype 1b | 3'UTR_16_ | NS3-383, NS3-418 |
| (no. of rules = 2) |  |  |
|  |  |  |
| Genotype 2a  (no. of rules = 8) | 3'UTR_133_ | NS5B-487 |
|  | 3'UTR_134_ | NS5B-156 |
|  | 3'UTR_146_ | NS2-52 |
|  | 3'UTR_151_ | NS2-12 |
|  | 3'UTR_151_ | NS5B-487 |
|  | 3'UTR_153_ | NS5B-487 |
|  | 3'UTR_158_ | NS2-12 |
|  | 3'UTR_165_ | NS2-12 |
|  |  |  |
| Genotype 2b | 5'UTR_203_ | NS3-274, NS3-358, NS3-384, NS3-555 |
| (no. of rules = 6) | 5'UTR_203_ | NS5B-186, NS5B-470 |
