# Supplementary material for: Novel Nucleotide and Amino Acid Covariation between the 5′UTR and the NS2/NS3 Proteins of Hepatitis C Virus: Bioinformatic and Functional Analyses
Source: PLoS One. 2011 Sep 28;6(9):e25530. doi: 10.1371/journal.pone.0025530 (PMC3182228; doi:10.1371/journal.pone.0025530)
Supplement: Table S3 — The frequencies (%) of co-evolutionary sites in the sampled HCV genome sequences. (DOCX) [file pone.0025530.s004.docx]

**Table S3.** **The frequencies (%) of co-evolutionary sites in the sampled HCV genome sequences.**

| Position | NS2 | | | | | |  | | NS3 | | |
| --- | --- | --- | --- | --- | --- | --- | --- | --- | --- | --- | --- |
|  | 14 | 41 | 76 | 110 | 211 | 212 | | 71 | | 175 | 621 |
| Genotype 1b (n=127) | | | | | | | | | | | |
| 5'UTR_243_G | F | I | I | I | G | Q | | I | | M | A |
| %^a^ | 79.5 | 84.3 | 87.4 | 89.7 | 65.4 | 85.0 | | 87.4 | | 92.1 | 73.2 |
| 5'UTR_243_A | L | L | V | L | S | K | | V | | L | T |
| %^a^ | 0.8 | 0.8 | 0.8 | 0.8 | 0.0 | 0.0 | | 0.0 | | 0.0 | 0.0 |
|  | | | | | | | | | | | |
| Genotype non-1b (n=90) | | | | | | | | | | | |
| 5'UTR_243_G | F | I | I | I | G | Q | | I | | M | A |
| %^a^ | 0.0 | 2.2 | 7.8 | 5.6 | 0.0 | 0.0 | | 0.0 | | 3.3 | 11.1 |
| 5'UTR_243_A | L | L | V | L | S | K | | V | | L | T |
| %^a^ | 73.3 | 81.1 | 73.3 | 80.0 | 75.6 | 77.8 | | 78.9 | | 77.8 | 78.9 |

^a^Number of site/Number of sampled HCV genome sequences according to genotype, expressed as percentage.
