# Supplementary material for: Novel Nucleotide and Amino Acid Covariation between the 5′UTR and the NS2/NS3 Proteins of Hepatitis C Virus: Bioinformatic and Functional Analyses
Source: PLoS One. 2011 Sep 28;6(9):e25530. doi: 10.1371/journal.pone.0025530 (PMC3182228; doi:10.1371/journal.pone.0025530)
Supplement: Table S4 — Oligonucleotides used for construction of replicon variants. (DOCX) [file pone.0025530.s005.docx]

**Table S4. Oligonucleotides used for construction of replicon variants.**

| Name | Wild-type sequences/  Primers used in site-directed mutagenesis reactions |
| --- | --- |
| NS2-14F/ NS2-F14L | 5'-gcggaggcgcggttttcgtaggtctgatact-3'/  5'-gcggaggcgcggttttagtaggtctgatact-3' |
|  |  |
| NS2-41I/  NS2-I41L | 5'-gctcatatggtggttacaatattttatcaccagggccga-3'/  5'-gctcatatggtggttacaatattttctcaccagggccga-3' |
|  |  |
| NS2-76I/ NS2-I76V | 5'-cgcgatccacccagagctaatctttaccatcacc-3'/  5'-cgcgatccacccagagctagtctttaccatcacc-3' |
|  |  |
| NS2-110I/ NS2-I110L | 5'-gcgcacacgggctcattcgtgcatgcatg-3'/  5'-gcgcacacgggctccttcgtgcatgcatg-3' |
|  |  |
| NS2-110I/ NS2-I110L (TTG) | 5'-gcgcacacgggctcattcgtgcatgcatg-3'/  5'-gcgcacacgggctcttgcgtgcatgcatg-3' |
|  |  |
| NS2-211G/ NS2-G211S | 5'-ccggcagacagccttgaagggcaggggtggc-3'/  5'-ccggcagacagccttgaaagccaggggtggc-3' |
|  |  |
| NS2-211G/ NS2-G211S (TCA) | 5'-ccggcagacagccttgaagggcaggggtggc-3'/  5'-ccggcagacagccttgaatcacaggggtggc-3' |
|  |  |
| NS2-212Q/ NS2-Q212K | 5'-cagccttgaagggcaggggtggcgact-3'/  5'-cagccttgaagggaaggggtggcgact-3' |
|  |  |
| NS3-71I/ NS3-I71V | 5'-cggcccaaagggcccaatcacccaaatgtac-3'/  5'-cggcccaaagggcccagtcacccaaatgtac-3' |
|  |  |
| NS3-175M/ NS3-M175L | 5'-gactttgtacccgtcgagtctatgggaaccact-3'/  5'-gactttgtacccgtcgagtctctgggaaccact-3' |
|  |  |
| NS3-621A/ | 5'-cccataaccaaatacatcatggcatgcatgtcggctgac-3'/ |
| NS3-A621T | 5'-cccataaccaaatacatcatgacatgcatgtcggctgac-3' |
